# Airway Mucosal Defense: Mucins, Innate Immunity, and Contemporary Mucoactive Strategies

**DOI:** 10.3390/biomedicines14040831

**Published:** 2026-04-06

**Authors:** Almira Akparova, Gaukhar Kurmanova, Gulzhakhan Omarova, Almagul Kurmanova, Moldir Zhunisbek, Magripa Bapaeva, Zhamilya Zhankina, Sholpan Sadykova, Amina Abdrakhmanova, Adema Samadin

**Affiliations:** 1 Faculty of Medicine and Healthcare, Al-Farabi Kazakh National University, Almaty 050040, Kazakhstan; akparova-a@yandex.kz (A.A.); gaukhar.kurmanova@kaznu.edu.kz (G.K.); sholpan.sadykova@kaznu.edu.kz (S.S.); amisaparova@gmail.com (A.A.); adema.samadin@gmail.com (A.S.); 2 City Clinical Hospital No 1, 2, Almaty 050006, Kazakhstan; moldirzhunisbek.kz@gmail.com (M.Z.); magripa.bapaeva@gmail.com (M.B.); 3 Department of Biology, Faculty of Natural Sciences, Friedrich-Alexander University, Erlangen-Nürnberg, Schlossplatz 4, 91054 Erlangen, Germany; zhamilya.zhankina@fau.de

**Keywords:** innate immunity, mucins, mucous membranes, pathogens, infections, microbiota, homeostasis, inflammation, immunological defense, MUC5AC, MUC5B, mucociliary clearance, mucoactive therapy

## Abstract

Mucins are highly glycosylated proteins that form the structural basis of mucus and represent a key component of innate immunity at mucosal surfaces, particularly in the respiratory tract. Beyond their mechanical barrier function, mucins actively participate in pathogen trapping, regulation of mucociliary clearance, modulation of inflammatory responses, and maintenance of epithelial homeostasis. Dysregulation of mucin synthesis, composition, or transport contributes to mucus hypersecretion, impaired airway clearance, and chronic inflammation in respiratory diseases such as asthma, chronic obstructive pulmonary disease, and cystic fibrosis. This review summarizes current insights into mucin biology, including their biosynthesis, structure, classification, and regulation, with emphasis on the gel-forming mucins MUC5AC and MUC5B. The role of mucins in mechanical protection, host–pathogen interactions, control of inflammation, and coordination of innate immune responses is reviewed. Attention is given to the interplay between mucins, immune cells, and microbial communities in maintaining airway barrier integrity. The article further examines mucoactive therapeutic strategies aimed at restoring mucus barrier function. Expectorants, mucolytics, mucoregulators, and mucokinetic agents are reviewed with respect to their mechanisms of action and clinical relevance. Established drugs, including N-acetylcysteine, carbocysteine, dornase alfa, ambroxol, and hypertonic solutions, are considered alongside emerging molecular targets such as NF-κB-dependent regulation of mucin expression, calcium-activated chloride channels, MARCKS-mediated mucin exocytosis, purinergic signaling pathways, and NO/cGMP signaling. Non-pharmacological approaches, including airway clearance techniques and respiratory rehabilitation, are covered concisely. Conclusions: Overall, this review highlights mucins as dynamic regulators of innate immunity and underscores the need for mechanism-based, personalized mucoactive therapies to improve outcomes in chronic inflammatory airway diseases.

## 1. Introduction

The innate immunity serves as the frontline defense mechanism against potentially harmful foreign biological elements, spanning from bacteria and viruses to fungi and parasites [1]. Over the past three decades, our understanding of its significance within the human body has undergone a profound shift, underscoring its pivotal role in immune defense. This reevaluation has highlighted its multifaceted contributions to maintaining homeostasis, orchestrating microbiota formation, and mediating various pathological processes.

The activation of innate immunity swiftly follows the entry of pathogens into the body, initiating responses within minutes to hours. Pathogen detection is performed by specialized pattern recognition receptors (PRRs), such as Toll-like receptors (TLRs), C-type lectin receptors (CLRs), Nod-like receptors (NLRs), and retinoid acid-inducible gene, RIG -(RIG-I)-like receptors (RLRs). Also important for activating the inflammatory response are NLRP1 proteins, a subtype of NLRs that can oligomerize and assemble into a complex known as the inflammasome [2]. Upon recognition, these receptors transmit crucial information through signaling pathways, orchestrating an immune response.

The disruption of innate immune function can have far-reaching consequences, ranging from inflammation and dysbiosis to immunodeficiency and autoimmune disorders [3]. Thus, understanding the intricate mechanisms governing innate immunity is paramount for devising effective strategies to combat infectious diseases and maintain overall health.

The components of innate immunity include physical barriers such as skin, mucous membranes, and mucus. There are various immune cells and proteins involved, as well as serum proteins associated with inflammation, pathogenic peptides, and receptors on immune cells that respond to inflammation mediators and release cellular responses. This multifaceted system is subdivided into cellular and humoral components. Immune cells such as natural killer cells, phagocytes, granulocytes, and select T and B lymphocyte populations form the cellular arm. Meanwhile, the humoral aspect encompasses biochemical components such as lysozyme, interferons, the complement system, and various inflammatory mediators [4,5].

The activation of innate immunity orchestrates a coordinated response, culminating in the recruitment of immune cells to the site of pathogen entry, detection, and subsequent clearance of invading pathogens. Notably, this activation primes the adaptive immune system for a tailored response [2].

At the forefront of innate immune defense lies the mucosal barrier, acting as the interface between the external environment and underlying tissues. Key to this defense are mucins, heavily glycosylated proteins abundant in mucus [6]. Mucins form a crucial first line of defense for mucous membranes against pathogens and environmental particles. Anchoring to epithelial villi and cilia of the respiratory tract, mucins create a protective barrier, further fortified by secretory mucins that aid in forming viscoelastic gels and retaining water [7]. Moreover, mucins play a vital role in cell signaling through interactions with specific receptors, known as glycan-binding proteins (GBPs), found on immune cells [8].

Current airway-protective therapies integrate pharmacological and non-pharmacological strategies. Pharmacological approaches predominantly involve mucoactive agents that modify mucus hydration, viscoelastic properties, composition, and clearance [9]. In addition to their classical effects on mucus rheology, various mucoactive drugs exert antioxidant, anti-inflammatory, and anti-infective actions, thereby directly influencing innate immune responses at the airway surface [10]. Non-pharmacological interventions, including airway clearance techniques and respiratory rehabilitation, further support mucociliary transport and mechanical removal of pathogens [11]. Collectively, modern therapeutic concepts emphasize that effective airway protection depends on maintaining the mucosal barrier as an integrated functional system, highlighting mucins as central targets for both established and emerging therapies.

## 2. Literature Search Strategy

A literature search was performed using the PubMed, Scopus, and Web of Science databases to identify publications related to airway mucins and mucus regulation. The search covered studies published from 2000 to January 2026 using combinations of the following keywords: mucins, MUC5AC, MUC5B, airway mucus, mucociliary clearance, airway inflammation, mucus hypersecretion, and mucoactive therapy. Only peer-reviewed articles published in English were considered. Original research articles, experimental studies, and relevant review papers focusing on the biology and clinical relevance of airway mucins were included. Conference abstracts, duplicate records, and studies not directly related to airway mucins were excluded. Approximately 210 publications were initially identified, and after screening for relevance, 95 studies were included and used to inform the narrative synthesis presented in this review.

## 3. Results

### 3.1. Mucins: Biosynthesis, Structure, and Classification

A total of 22 human mucin genes have been identified [12]. These genes are now universally denoted by the abbreviation ‘MUC’ followed by a serial number corresponding to the specific protein. Primarily synthesized by superficial epithelial goblet cells, as well as mucous and serous cells within the submucosal glands, mucins exhibit notable variations in type and quantity across different bodily locations [12].

The significance of these glycoproteins is underlined by the substantial energy expenditure required by mucosal cells for mucin production, a demand that escalates significantly during infections [13]. Apomucins, the building blocks of mucins, are composed of specific peptides featuring two discernible regions: a central serine–threonine–proline-rich tandem repeat region (STP tandem repeats), serving as the primary site for O-linked mucin-type glycosylation [14], and carboxy- and amino-terminal non-terminal repeat regions [15]. The intricate process of assembling these complex structures involves the formation of a “bottlebrush” configuration, meticulously arranged around a core strand of mucin protein. Carbohydrates, predominantly comprising N-acetylglucosamine (GlcNAc), fucose (Fuc), galactose (Gal), and sialic acids (N-acetylneuraminic acid (NeuNAc)), form the backbone of these complex oligosaccharide structures. Additionally, trace amounts of mannose and sulfate are incorporated within these structures. Oligosaccharide chains, varying in length from 2 to 20 monosaccharides, can adopt linear or branched structures [13]. In secreted mucosal proteins, the terminal regions are enriched with cysteines and harbor several conserved domains, albeit with comparatively lower levels of glycosylation [16].

**Figure 1:**
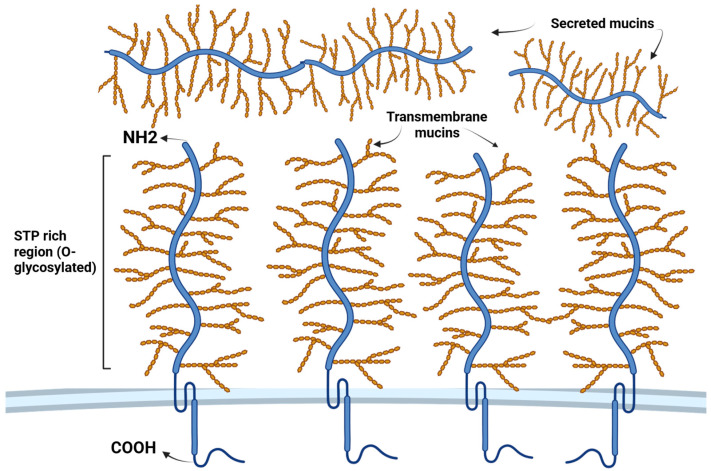
Structural organization of transmembrane and secreted mucins. Created in BioRender. Akparova, A. (2026) https://BioRender.com/fe2zo1c (accessed on 19 March 2026).

To date, researchers have identified more than 20 distinct mucins, with at least 8 being expressed in the respiratory tract. These mucins are categorized into three classes: secreted and non-polymerizable (such as MUC7), secreted and polymerizable (including MUC5AC and MUC5B), and transmembrane (which includes MUC1, MUC4, MUC16, and MUC20) [12] (Figure 1). In the lungs, the predominant secreted mucins are MUC5AC and MUC5B, constituting approximately 90% of the mucin content in sputum [8]. Furthermore, various other mucins, such as MUC2, MUC6, MUC8, MUC13, MUC19, MUC21, and MUC22, are found in the human lower respiratory tract [17].

The activation of mucin expression occurs in goblet cells in response to various stimuli, including cytokines, proteases, bacteria, and hazardous pollutants, either at the transcriptional or post-transcriptional level, and is mediated by various intracellular cascades [18]. Mucin expression is tightly regulated at multiple levels, including transcriptional, post-transcriptional, and epigenetic regulation. Post-translational modifications play a crucial role in mucin function, facilitating binding interactions and the removal of microbes and contaminants [12].

The assembly, storage, and secretion of polymeric mucins involve a complex sequence of steps within various intracellular and extracellular compartments. Following nuclear transcription, the translation of secreted mucins occurs perinuclearly on ribosomes associated with the rough endoplasmic reticulum. Dimerization occurs in carboxy-terminal domains, followed by glycosylation initiated by O-linked N-acetylgalactosamine and subsequent polymerization at the cysteine-rich amino group and carboxy-terminal domains [19,20]. In the Golgi complex, this process continues through the binding of various mucin saccharides, the folding of cysteine-rich N- and C-terminal domains, and further N-glycosylation and C-mannosylation, which began in the endoplasmic reticulum. Notably, the expression profile of mucins varies depending on the tissue type, with the gastrointestinal tract exhibiting the highest and most diverse expression levels [13].

### 3.2. The Role of Mucins in Innate Immune Defense

**Figure 2:**
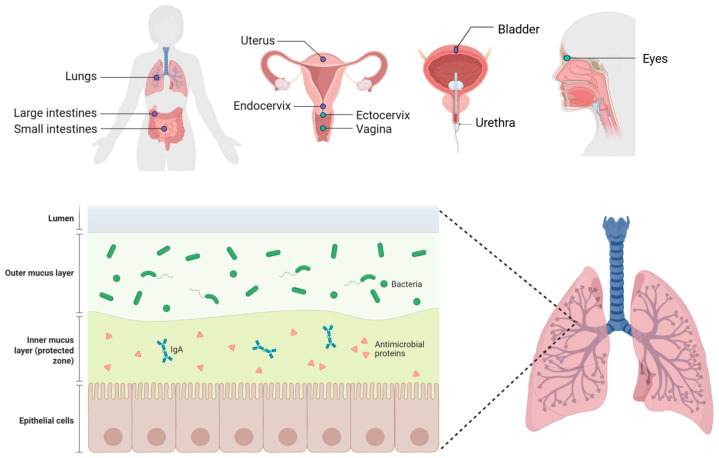
Organization of the mucosal barrier across mucosal surfaces. Created in BioRender. Akparova, A (2026) https://BioRender.com/fe2zo1c (accessed on 19 March 2026).

Mucins play a vital role in the immunity of the mucous membranes of the eyes and the respiratory, reproductive, and urinary tracts, as well as the gastrointestinal tract [21] (Figure 2). All these tissues are covered with microflora, which vary in their composition and concentration. Mucosal epithelia secrete mucin glycoproteins in copious amounts, while cell surface mucins adorn the apical glycocalyx of all mucosal epithelia, defining their characteristic features [21].

A layer of viscous mucus blankets the surfaces of mucous membranes, exhibiting varying thicknesses from 10 microns in the eyes and trachea to 300 microns in the stomach and 700 microns in the intestines [22,23,24]. This mucus, presented in a gel-like form, plays multifaceted roles beyond mere physical barrier and lubrication functions. It acts as a matrix housing an array of antimicrobial molecules, which are crucial for trapping and neutralizing pathogens [25]. In the eye, mucus contributes to lubrication and cleansing, while in the respiratory tract, it aids in capturing infectious agents, facilitating their clearance through mechanisms such as mucociliary transport or coughing. Additionally, mucus maintains airway moisture and actively participates in immune responses [25]. In the digestive tract, mucins are part of mucus, which forms “biofilm” structures that isolate harmful substances from direct contact with epithelial cells. The interplay between mucin glycoproteins and water generates a diffusion barrier essential for immune regulation within the digestive tract, ensuring homeostasis [18]. Moreover, cervical and vaginal mucins extend their protective repertoire, offering lubrication and antimicrobial defense and enhancing sperm motility within the mucus environment [26]. Thus, mucins stand as integral components, orchestrating immune defense and maintaining homeostasis across diverse mucosal surfaces.

### 3.3. Mechanical Protection of Mucous Membranes

The involvement of mucins in the defense against foreign or harmful substances is closely tied to their role in the provision of the mechanical [27] and physicochemical characteristics of mucus [13,27]. Mucins construct a selectively permeable sticky mesh filter that regulates the diffusion of particles and small molecules through several mechanisms. This includes physically impeding particles larger than the mesh openings and selectively controlling molecule passage through interactions with charged and hydrophobic residues in mucin polymers [28]. Notably, positively charged molecules have a stronger affinity for negatively charged mucin [29]. Furthermore, the arrangement of charges and other chemical properties, along with the presence of hydrophobic elements on the molecule, influences these interactions [29,30,31].

**Figure 3:**
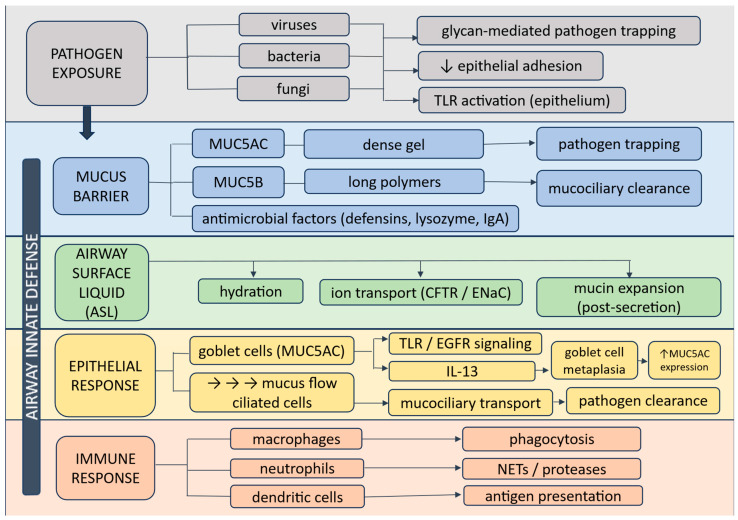
Multilayer airway innate defense against inhaled pathogens. Colors indicate functional layers: mucus barrier (blue), airway surface liquid and ion transport (green), epithelial responses (yellow), and immune response (beige). Arrows represent functional interactions and regulatory pathways between components, while upward arrows indicate increased expression or activity.

The effective clearance of mucus is crucial for airway defense, acting as the primary barrier against harmful particles [32,33] (Figure 3).

The airway barrier consists of an integrated system involving mucus (MUC5AC and MUC5B), mucociliary clearance, epithelial sensing, and immune cell responses. This coordinated network enables pathogen trapping, transport, and clearance while regulating inflammation.

Optimal mucociliary clearance hinges upon both the quality and quantity of mucins produced, dictating the viscoelastic properties of mucus. Viscoelasticity, the amalgamation of viscous (resistance to flow) and elastic (restoration to original shape) characteristics, underpins this process. Contemporary insights into mucociliary clearance propose its operation through a high-viscosity gel layer (mucus) overlaying a low-viscosity sol, termed the periciliary liquid (PCL) [34,35,36]. The gel layer, harboring entrapped foreign particles, is expelled from the airways via the rapid and coordinated beating of cilia within the PCL. Membrane-bound mucins and other high-molecular-weight glycoconjugates in the PCL prevent mucins from the gel layer from permeating the interciliary space, thus upholding its low-viscosity state and stabilizing mucociliary transport [7]. Every component of the ciliary clearance system—mucus, PCL, and cilia—is indispensable for its normal function; any aberration in one element can precipitate severe airway dysfunction and disease [17].

Mucociliary clearance and barrier fortification primarily hinge on mucins MUC5AC and MUC5B, the principal gel-forming mucins in the airways. While MUC5AC may foster general mucociliary clearance, MUC5B might assume greater importance in combating specific pathogens or airway irritants [37].

Although both MUC5AC and MUC5B are the principal gel-forming mucins of airway mucus, accumulating experimental and clinical evidence suggests that they perform partially distinct and sometimes opposing functions in airway defense. A comparative analysis of experimental and clinical studies suggests that MUC5B is primarily involved in maintaining baseline mucociliary clearance and antimicrobial defense [38,39,40], whereas MUC5AC is more closely associated with inflammatory responses and pathological mucus hypersecretion [41,42,43].

Several experimental studies highlight the critical role of MUC5B in physiological airway protection. In murine models, Muc5b is the dominant gel-forming mucin in the airways and is essential for normal mucociliary clearance. Mice lacking Muc5b develop severe defects in airway defense, including the accumulation of aspirated material, chronic bacterial infections, airway obstruction, and inflammation, indicating that MUC5B plays a central role in maintaining airway sterility and mucus transport mechanisms [44,45]. These findings are supported by clinical observations demonstrating that the congenital absence of MUC5B in humans results in impaired mucociliary clearance and chronic respiratory disease [46].

In contrast, MUC5AC expression appears to be more closely linked to inflammatory airway remodeling and mucus hypersecretion in chronic airway diseases [42,47]. Multiple studies consistently report increased MUC5AC expression in asthmatic individuals, where it is primarily produced by goblet cells of the surface epithelium [42]. Elevated MUC5AC levels in sputum have been associated with type-2 airway inflammation and disease exacerbations, suggesting that excessive MUC5AC production may contribute to pathological mucus plugging and airflow limitations.

However, the relative contributions of MUC5AC and MUC5B remain a subject of debate. Some studies report increased levels of both mucins in airway secretions of patients with asthma and COPD [48,49], while others demonstrate that MUC5B is the predominant mucin in healthy individuals and that disease states are characterized mainly by a shift toward increased MUC5AC levels [41,42] and a decreased expression of MUC5B [46]. This shift in the MUC5AC/MUC5B ratio may represent an important pathogenic mechanism, as higher ratios have been associated with eosinophilic inflammation and more severe airway disease. Evidence from studies in smokers and patients with COPD further supports the concept of the differential regulation of these mucins [49]. Smoking exposure has been positively associated with increased MUC5AC expression in goblet cells, whereas MUC5B expression tends to decrease, suggesting distinct regulatory pathways and potentially different biological functions. Moreover, lower MUC5B expression has been linked to structural airway abnormalities and emphysema development, while higher MUC5AC levels correlate with disease progression and airflow limitations [47].

These apparently conflicting findings may partly reflect differences in the study design, disease phenotypes, and methodological approaches used to quantify mucins (e.g., gene expression, protein levels, or glycoform analysis). Additionally, mucin glycosylation patterns can substantially alter mucus rheology and interactions with pathogens, further complicating direct comparisons between studies.

Taken together, the current body of evidence suggests that MUC5B functions primarily as a key component of physiological airway defense and mucociliary clearance, whereas MUC5AC may play a more prominent role in inflammatory mucus hypersecretion and airway obstruction during disease. Rather than acting redundantly, these mucins appear to perform complementary functions, and the disruption of their normal balance may contribute to impaired airway defense in chronic respiratory disorders.

Furthermore, mucus overproduction significantly contributes to morbidity and mortality related to respiratory ailments [50]. Infectious agents and inflammatory mediators activate the expression of mucin genes, leading to mucin overproduction via goblet cell hyperplasia and goblet cell metaplasia. Proteomic analyses of respiratory mucus unveil the close association of mucins with other proteins that boast antimicrobial, antiprotease, antioxidant, and anti-inflammatory properties [51,52]. The coupling of mucins with these bioactive molecules within secretory granules before exocytosis facilitates their enhanced interaction with invading pathogens post-exocytosis [53]. Among the molecules binding to mucins are trefoil factors (TFFs), which regulate mucosal viscosity and potentially bolster the protective capacity of the respiratory mucosal barrier [54,55].

In preserving the respiratory tract, MUC5AC and MUC5B play pivotal roles by impeding the epithelial adhesion and cytotoxicity of pathogens via direct interactions with infectious agents through glycan structures [56]. Additionally, mucins partake in innate immune function by directly engaging with dendritic cells, with mucin-secreting cells functioning as sentinels that detect environmental threats to the epithelial surface [12]. The significance of murine Muc5b in airway immunity is underscored by studies that demonstrate its necessity for mucociliary clearance and the adverse consequences of Muc5b deficiency, including airway obstruction, heightened infection risk, inflammation, and impaired macrophage phagocytosis and viability [44].

Airway mucus functions not only as a physical barrier but also as a dynamic ecological niche supporting a diverse respiratory microbiome. In healthy lungs, mucins—particularly MUC5B and MUC5AC—contribute to microbial spatial organization and clearance, thereby maintaining host–microbiota homeostasis [57,58]. Mucin glycans serve as a selective nutrient source for specific microbial taxa, promoting metabolic cooperation and stability within the microbial community [59].

Disruption of mucus properties, including hyperconcentration and altered glycosylation, leads to microbial dysbiosis characterized by reduced diversity and enrichment of pathogenic species [60,61]. In parallel, pathogens actively exploit mucin biology: Pseudomonas aeruginosa enhances mucin production and adhesion through glycan interactions, while respiratory viruses utilize sialylated mucins and neuraminidase activity to penetrate the mucus barrier.

Importantly, mucin composition influences microbial behavior. MUC5B supports effective mucociliary clearance and microbial homeostasis, whereas increased MUC5AC is associated with mucus stasis and pathogen persistence. These interactions highlight mucus as an active regulator of the airway microbiome. However, the mechanisms linking mucin composition to microbial community structure remain incompletely understood, thus representing an important area for future research.

### 3.4. Regulation of Inflammation

Mucins, the gel-forming proteins ubiquitous in mucous membranes, emerge as dynamic orchestrators of inflammation regulation, immune response modulation, and epithelial repair facilitation. The capacity of gel-forming mucins to capture, retain, and release bioactive molecules [62] allows them to regulate inflammation and immune responses, as well as influence epithelial repair after injury. Notably, mucins reversibly bind an array of molecules, including cytokines (such as IL-1, IL-4, IL-6, and IL-7), growth factors, and trefoil factors (TFFs) [17]. In the airway epithelium, MUC1 has immunomodulatory properties; it is expressed in macrophages and reduces the phagocytosis and release of proinflammatory cytokines by macrophages [63]. Insights from studies, such as that by Singanayagam et al., underscore the impact of mucins on inflammation modulation. Muc5ac-deficient mice exhibited attenuated rhinovirus-induced airway inflammation, while exogenous administration of MUC5AC glycoprotein intensified inflammatory responses and heightened extracellular adenosine triphosphate release [64]. Meanwhile, MUC13 enhances NF-κB activation, a master transcription factor governing the expression of immune response, apoptosis, and cell cycle genes [65].

The airway mucins interact dynamically with the respiratory microbiota. Mucin glycans provide binding sites and nutrient sources for microorganisms, influencing microbial colonization and community structure. At the same time, mucin gels limit epithelial adhesion of pathogens and facilitate their removal through mucociliary clearance. The microbial components can also regulate mucin production through epithelial signaling pathways such as Toll-like receptors, highlighting the bidirectional relationship between airway microbiota and mucin biology [66,67].

The differential biological roles of MUC5AC and MUC5B also have important implications for the clinical effectiveness of mucoactive therapies. Most currently used mucoactive drugs—including mucolytics, mucoregulators, and mucokinetic agents—primarily modify the physical properties of airway secretions rather than directly targeting mucin biosynthesis or secretion pathways. Consequently, their therapeutic benefit may depend on the underlying mucin composition and disease phenotype. In healthy airways, where MUC5B is the predominant mucin and supports efficient mucociliary clearance, excessive suppression of mucus production could theoretically impair host defense. In contrast, inflammatory airway diseases such as asthma are often characterized by increased MUC5AC expression and an altered MUC5AC/MUC5B ratio, which promotes the formation of adhesive mucus plugs and impaired mucus transport. In such contexts, therapies that reduce mucus hypersecretion or modify mucus viscoelasticity may restore airway patency without compromising mucosal immunity.

However, clinical responses to mucoactive therapy remain heterogeneous, partly because most treatments do not distinguish between protective and pathological components of airway mucus. This limitation may explain why the clinical efficacy of many mucoactive agents is modest and varies across respiratory diseases. Increasing evidence suggests that effective treatment strategies should consider mucin-specific mechanisms and patient phenotypes, rather than applying uniform mucus-modifying approaches. Integrating mucin biology with clinical stratification may therefore represent an important step toward precision medicine in airway diseases.

Given the central role of mucins in airway defense, therapeutic strategies are increasingly shifting from nonspecific mucus modification toward targeting the underlying mechanisms of mucin regulation. This approach reflects the growing recognition of mucin heterogeneity and supports the development of phenotype-driven therapies in airway diseases.

### 3.5. Mucoactive Strategies in Airway Protection

Mucoactive agents comprise a broad class of therapies designed to alter the production, composition, viscoelastic characteristics, and clearance of airway mucus. Based on their primary mechanisms of action, these drugs are commonly categorized as expectorants, mucolytics, mucoregulators, and mucokinetic agents (Table 1). Although they differ in their modes of action, all these agents, to varying extents, interact with the structure of the mucin barrier and can thereby modulate the effectiveness of innate immune defenses.

#### 3.5.1. Expectorant Agents

The impaired hydration and the increased viscosity of bronchial secretions are key factors that limit the effectiveness of mucociliary clearance and the cough reflex in respiratory diseases. The expectorant agents are used to increase the volume and/or aqueous fraction of airway secretions, thereby facilitating their removal and improving pulmonary ventilation. These drugs generally enhance coughing or sneezing, promoting the movement of mucus from the lower to the upper airways, where it can be expelled. As a result, the expectorants may improve alveolar ventilation and reduce inflammation associated with the unfavorable mechanical properties of respiratory secretions [9].

Among the primarily used expectorant therapies are hypertonic solutions administered as aerosols, including saline, urea, ascorbic acid, and related compounds. These substances act as osmotic agents, increasing water influx into the mucus gel and expanding secretion volume, which facilitates sputum formation and clearance [68].

The inhalation of *hypertonic saline solutions* (typically 3–7%) enhances osmotic hydration of the mucin gel, disrupts intermolecular interactions, and increases the mobility of mucin polymers. This approach has demonstrated clinical efficacy in patients with cystic fibrosis and is widely employed for the induction of diagnostic sputum samples. However, the therapeutic effect of hypertonic solutions may be less pronounced in conditions that are not associated with significant disturbances in ion transport [9].

Another osmotic agent that has undergone clinical evaluation is *mannitol*, a sugar alcohol that, when inhaled as a dry powder, draws water and secretions into the airways, thereby promoting their hydration [69].

*Guaifenesin* (glyceryl guaiacolate) is a well-established expectorant that is thought to activate cholinergic pathways. It reduces surface tension and mucus viscosity and may stimulate the secretion of less viscous bronchial fluid. Although the precise mechanisms by which guaifenesin affects mucin structure remain insufficiently characterized, involvement of vagally mediated reflexes and enhanced exocytosis of serous components has been proposed.

Preclinical studies have demonstrated that guaifenesin can improve mucociliary clearance; however, its clinical efficacy remains a subject of ongoing debate [70].

Trinucleotide compounds, such as uridine triphosphate and adenosine triphosphate, modulate ion transport via P2Y_2_ receptors. Inhaled uridine triphosphate has been shown to enhance mucociliary clearance in healthy volunteers, supporting the potential of this pathway for pharmacological stimulation of airway secretion hydration [71].

#### 3.5.2. Mucolytic Agents

Conventional mucolytics modify the physicochemical properties of mucus by cleaving disulfide bonds that link individual mucin monomers [72]. Free thiol groups can disrupt these bonds through interactions with cysteine residues located within the protein backbone of mucins. Because disulfide bridges are critical for maintaining the structural stability of complex glycoproteins such as mucins, cysteine residues represent a primary molecular target for thiol-containing compounds. The most widely recognized agent in this class is N-acetylcysteine (NAC).

NAC has been used in clinical practice for more than eight decades and is among the most extensively investigated mucolytic drugs. Its molecule contains a single free thiol (sulfhydryl) group, which underlies its principal pharmacological actions. The mucolytic activity of NAC is mediated by the disruption of disulfide linkages between mucin polymers, resulting in mucin depolymerization and a reduction in the viscosity of the mucus gel.

In addition to its mucus-modifying properties, NAC exhibits anti-inflammatory, antioxidant, and anti-infective effects. It decreases the generation of reactive oxygen species (ROS) and attenuates the production of inflammatory mediators that contribute to the development of respiratory dysfunction [73].

Enzyme-derived mucolytics include dornase alfa, a recombinant human deoxyribonuclease that degrades extracellular DNA within the airways, leading to decreased sputum viscosity and improved mucus clearance from the lungs [74]. In cystic fibrosis, neutrophil degradation in infected airways is accompanied by the release of large amounts of DNA (known as neutrophil extracellular traps (NETs)), which markedly impairs the rheological properties of airway secretions and disrupts mucociliary transport [75]. By reducing both the size and concentration of DNA in sputum, dornase alfa directly alleviates bronchial obstruction, providing a pathogenetic rationale for its clinical use [74,75].

Clinical trials have demonstrated that treatment with dornase alfa results in significant improvements in pulmonary function parameters in patients with cystic fibrosis [76]. Moreover, its use is associated with a reduced incidence of exacerbations and infections requiring intravenous antibiotic therapy [76]. Further direct effects on host defense mechanisms, such as the release of antimicrobial peptides and the degradation of extracellular DNA involved in bacterial biofilm formation and the persistence of neutrophilic inflammation [77], could be linked to these benefits, further contributing to enhanced mucociliary clearance and a reduction in the bacterial burden in the lower airways [78].

Dornase alfa has been shown to lower levels of inflammatory markers and neutrophil-associated metalloproteinases in cystic fibrosis, suggesting a potential advantage of initiating therapy at earlier stages of the disease. Although in routine clinical practice the drug is primarily prescribed to patients with advanced lung involvement, data from both interventional and observational studies indicate that early initiation of dornase alfa therapy may slow the decline in lung function and reduce exacerbation risk, thereby potentially influencing long-term disease outcomes [74].

#### 3.5.3. Mucoregulatory Agents

Mucoregulators constitute a pharmacological class of agents capable of modifying both the qualitative and quantitative composition of mucins, as well as modulating inflammatory pathways that underlie goblet cell hyperplasia and airway mucosal remodeling. This group includes carbocysteine, anticholinergic drugs, glucocorticoids, and macrolide antibiotics; however, their mechanisms of action differ substantially. In contrast to agents that primarily act through neural regulation or immune modulation, carbocysteine exerts a direct effect on the biochemical properties of mucus and on oxidative stress-related processes.

Carbocysteine (S-carboxymethyl-L-cysteine; SCMC or SCMC-Lys) is a thiol derivative of L-cysteine and one of the most extensively studied mucoregulatory agents widely used in the management of chronic obstructive pulmonary disease. It exhibits pronounced antioxidant and anti-inflammatory activity [79]. Its principal mechanism involves restoration of the physiological balance between sialomucins and fucosylated mucins, a ratio that is critical for maintaining optimal rheological characteristics of the mucin gel.

Carbocysteine promotes the synthesis of low-viscosity sialomucins while simultaneously suppressing the production of highly viscous mucins, thereby improving mucus architecture, increasing elasticity, and reducing adhesiveness [80]. In addition, through its carboxymethyl moiety, carbocysteine interacts with mucin disulfide bonds, enhancing the viscoelastic properties of airway secretions and improving the efficiency of mucociliary clearance.

Experimental and clinical studies have demonstrated that carbocysteine modulates mucus production by reducing the imbalance between MUC5B and MUC5AC and by restoring MUC5B protein levels in COPD [81]. Moreover, its effects extend to ion transport mechanisms: investigations involving the CFTR channel have shown increased efflux of chloride ions and glutathione, which further contributes to improved hydration of the mucus layer [82].

Anticholinergic agents reduce mucus secretion by blocking M3 muscarinic receptors in submucosal glands, thereby inhibiting parasympathetic stimulation of secretory activity. Inhaled anticholinergics, such as atropine or ipratropium, diminish pathological mucus hypersecretion without significantly altering the normal volume or viscosity of airway secretions [83]. Their clinical use, however, may be limited by adverse effects.

Glucocorticoids, including prednisolone, exert only a modest influence on mucociliary clearance, whereas macrolide antibiotics (e.g., azithromycin and clarithromycin) can reduce sputum production, potentially via immunomodulatory mechanisms [84,85].

#### 3.5.4. Mucokinetic Agents

Mucokinetic drugs constitute a subgroup of mucoactive agents whose primary action is to facilitate mucus elimination from the airways by stimulating cough and enhancing mucociliary transport. Their effects are mediated through increased ciliary beat frequency and synchronization, augmentation of expiratory airflow, and reduction in adhesive interactions between mucus and the airway epithelium. Despite their theoretical benefits, the clinical efficacy of mucokinetic agents in chronic pulmonary disorders remains limited.

Mucokinetics, referred to as cough stimulants, promote bronchial secretion clearance through various mechanisms, including enhancement of ciliary motility and/or activity, intensification of airflow during coughing, and attenuation of mucus–cilia adhesion within the respiratory tract. However, available evidence indicates that these agents provide only modest improvement in airway clearance in patients with chronic lung diseases [9].

The class of mucokinetic agents includes bronchodilators, surfactant-active substances, and certain mucolytics that possess additional kinetic properties.

Bronchodilators are conventionally regarded as airway-expanding drugs; however, they are also classified as mucokinetic, mucoactive agents [9]. β_2_-adrenergic agonists are capable of increasing ciliary beat frequency and enhancing expiratory airflow, thereby facilitating mucus transport and clearance.

Previous studies have demonstrated that the secretory activity of serous cells in submucosal airway glands may be partially restored in cystic fibrosis with salmeterol, a β_2_-adrenergic agonist [86]. Furthermore, this therapeutic approach has been shown to improve mucociliary clearance in patients with cystic fibrosis who exhibit airway reversibility [87].

Nevertheless, bronchodilators may have variable effects on airway secretion dynamics and airway stability. In certain conditions, including bronchomalacia and small airway disease, excessive smooth muscle relaxation may contribute to airway collapse. Emerging experimental evidence suggests that airway smooth muscle tone may exert a stabilizing effect on the airway tree, and that excessive bronchodilation may, under some circumstances, promote small airway closure and ventilation heterogeneity [88].

Given that increased expiratory airflow can enhance cough efficiency, bronchodilators are more appropriately considered adjuncts to cough-mediated airway clearance rather than direct mucokinetic agents [89].

Among mucokinetic drugs, ambroxol occupies a distinctive position. Similar to N-acetylcysteine, it modulates mucus secretion and exerts anti-inflammatory effects, particularly when administered at higher doses. Ambroxol stimulates mucus production while simultaneously reducing its viscosity, thereby facilitating more effective clearance of airway secretions [90].

Ambroxol enhances serous secretion, increases fluid penetration into the airways, and liquefies dense mucus and sputum. In addition, it promotes surfactant synthesis, stimulates ciliary activity, and facilitates expectoration [91]. Through these mechanisms, ambroxol improves the rheological properties of airway secretions and accelerates mucin layer transport.

#### 3.5.5. Emerging Directions in the Development of Novel Mucoactive Therapies

Mucus hypersecretion and excessive expression of gel-forming mucins—most notably MUC5AC—represent core pathogenic mechanisms in chronic inflammatory airway diseases. Therefore, contemporary mucoactive strategies concentrate on molecular control of mucin synthesis and secretion, suppression of inflammatory signaling cascades that promote goblet cell metaplasia, and reducing mucus viscosity.

*Lipid mediators and neurogenic pathways*

11,12-Epoxyeicosatrienoic acids (11,12-EETs), generated from arachidonic acid via CYP epoxygenase activity, have demonstrated pronounced anti-inflammatory effects through modulation of the NF-κB signaling pathway. This activity is associated with reduced mucus production and attenuation of pulmonary inflammation [92]. Pharmacological induction of CYP epoxygenases using fibrate-like compounds is therefore being studied as a promising approach for correcting mucin hypersecretion.

Neurogenic regulation of mucus secretion is mediated by tachykinins acting through NK1 and NK2 receptors. Despite strong preclinical evidence, NK receptor antagonists such as FK224 and CP99994 have failed to demonstrate clinically meaningful efficacy in controlling mucus hypersecretion [93], which has limited their further development.

*Modulation of NO/cGMP signaling and ion channel activity*

Sildenafil, a phosphodiesterase-5 inhibitor, has been shown to suppress acrolein-induced airway inflammation, goblet cell metaplasia, and MUC5AC expression via activation of the nitric oxide/cGMP signaling pathway [94]. Given its well-characterized safety profile, this class of compounds is of interest for potential drug repurposing as mucoregulatory agents.

Calcium-activated chloride channel proteins (CLCA-1) are markedly overexpressed in chronic obstructive pulmonary disease, asthma, and cystic fibrosis. Pharmacological inhibition of CLCA-1, most notably with niflumic acid, effectively reduces mucin expression in airway epithelial cells and human lung tissue [95,96], positioning CLCA-1 as one of the most well-substantiated molecular targets in modern mucoactive therapy.

*Promising signaling targets: P2Y_2_, MARCKS, and RAR-α*

Activation of the purinergic P2Y_2_ receptor by ATP and UTP stimulates mucus secretion [97]. Although clinical studies are currently lacking, the development of P2Y_2_ antagonists remains a potentially valuable therapeutic direction.

The myristoylated alanine-rich C-kinase substrate (MARCKS) protein plays a central role in mucin exocytosis. MARCKS-associated peptides have demonstrated the ability to suppress mucus hypersecretion in vivo [98] and are considered among the most promising candidates for targeted mucoregulatory therapy.

Mucin expression is also regulated through the retinoic acid receptor RAR-α. While RAR-α antagonists could theoretically reduce mucin production, clinical evidence supporting this approach is currently unavailable [99].

Collectively, current evidence suggests that the greatest therapeutic potential lies in mucoactive agents targeting NF-κB-dependent regulation of MUC5AC, as well as pathways involving CLCA-1, MARCKS, and NO/cGMP signaling.

#### 3.5.6. Non-Pharmacological Mucoactive Strategies

Modern respiratory rehabilitation programs, including kinesitherapy and respiratory therapy techniques (such as inhalation therapy, positive expiratory pressure, and high-frequency oscillation), aim to enhance airway drainage and optimize the mucosal barrier. However, analysis of contemporary studies reveals substantial heterogeneity in applied methodologies, outcome measures, and intervention frequency, complicating cross-study comparisons. The absence of unified international guidelines for the selection of mucoactive and rehabilitative approaches underscores the need for standardized treatment protocols that account for the pathophysiology of mucin-mediated airway defense, patient age, and disease phenotype [11].

#### 3.5.7. Clinical Effectiveness and Limitations of Mucoactive Therapies

The clinical efficacy of mucoactive therapies remains inconsistent across respiratory diseases, reflecting both heterogeneity of clinical trial design and fundamental differences in mucus composition and pathophysiology. Although mucoactive agents are intended to reduce mucus viscosity and improve clearance, their clinical impact is generally modest. A Cochrane meta-analysis demonstrated only a small reduction in exacerbation frequency, without consistent improvements in lung function, quality of life, or mortality [100], indicating that mucus modification alone is insufficient to substantially alter disease outcomes.

In COPD, thiol-based mucolytics such as N-acetylcysteine, carbocysteine, and erdosteine may reduce exacerbation frequency in selected patients, particularly those with the chronic bronchitis phenotype and frequent exacerbations [101,102,103]. However, their effectiveness varies across studies and populations. Erdosteine appears to provide more consistent reductions in exacerbation risk and duration [100,101], whereas the effects of N-acetylcysteine and carbocysteine are influenced by concomitant inhaled corticosteroid use and underlying inflammatory phenotype [102]. Importantly, recent trials using high-dose N-acetylcysteine have failed to demonstrate significant clinical benefit [104]. Consistent with these findings, current GOLD and ATS/ERS recommendations consider mucolytics as optional add-on therapy in carefully selected COPD patients, emphasizing heterogeneity of response and the need for individualized treatment strategies [103,105].

In bronchiectasis, high-quality evidence does not support the use of mucoactive agents as a standalone therapy. A large randomized controlled trial demonstrated that neither hypertonic saline nor carbocysteine significantly reduced exacerbations over 52 weeks [106]. These results highlight a discrepancy between physiological effects on mucus rheology and clinically meaningful outcomes. Accordingly, ERS guidelines recommend that mucoactive agents should be used as part of a comprehensive airway clearance program, rather than as isolated pharmacological interventions [107]. This underscores the importance of integrating pharmacological and mechanical approaches to achieve effective mucus removal.

In cystic fibrosis, conventional thiol-based mucolytics have not demonstrated consistent improvements in lung function, exacerbation rates, or quality of life [108,109], whereas dornase alfa has shown clear clinical benefit [110], likely due to its ability to target extracellular DNA rather than mucin polymers. Moreover, current CF management strategies emphasize disease-modifying treatments such as CFTR modulators, which restore ion transport and airway surface hydration, thereby addressing the underlying mechanism of mucus dysfunction rather than its secondary rheological properties [111,112].

In asthma, there is no evidence supporting the routine use of mucolytics in current clinical guidelines. GINA recommendations do not include mucoactive agents as standard therapy, reflecting the lack of randomized clinical evidence demonstrating efficacy [100]. Instead, therapies targeting type 2 inflammation, including biologics directed against IL-4/IL-13 and IL-5 pathways, have shown the ability to reduce mucus hypersecretion and airway obstruction, indicating that mucus abnormalities in asthma are primarily driven by inflammatory mechanisms rather than intrinsic alterations in mucus structure [113,114,115,116].

Importantly, these inconsistencies in clinical efficacy may be partially explained by the differential biological roles of major airway mucins. MUC5B is primarily associated with physiological mucociliary clearance and antimicrobial defense, whereas MUC5AC is linked to inflammatory mucus hypersecretion and airway obstruction. Non-selective mucoactive therapies do not distinguish between these functionally distinct mucin pools and may therefore reduce both protective (MUC5B-dominant) and pathological (MUC5AC-enriched) mucus components. This lack of specificity may attenuate clinical benefit and, in some cases, potentially impair host defense.

Taken together, current evidence and guideline recommendations consistently indicate that mucoactive therapies should not be applied uniformly across respiratory diseases. Instead, their use should be integrated into a phenotype-driven framework that considers mucin composition, inflammatory endotype, and disease-specific mechanisms. Future therapeutic strategies should therefore focus on selective modulation of mucin subtypes, combination approaches with airway clearance techniques, and alignment with precision medicine principles.

## 4. Conclusions

Mucins represent a central structural and functional component of the innate immune system at mucosal surfaces, particularly within the respiratory tract, where they integrate mechanical protection, pathogen trapping, immune modulation, and regulation of epithelial homeostasis. Far from being passive barrier molecules, mucins actively participate in host defense through highly regulated biosynthesis, complex glycosylation patterns, and dynamic interactions with immune cells, microbiota, and inflammatory mediators. Disruption of mucin composition, hydration, or clearance profoundly alters mucosal barrier integrity and contributes to the pathogenesis of chronic inflammatory airway diseases.

This review highlights that effective airway defense depends not only on the quantity of mucus produced but also critically on its qualitative properties, including viscoelasticity, hydration, and coordinated transport by the mucociliary apparatus. Imbalances in gel-forming mucins, most notably MUC5AC and MUC5B, along with goblet cell hyperplasia and impaired mucociliary clearance, underlie mucus obstruction, chronic infection, and persistent inflammation observed in diseases such as COPD, asthma, and cystic fibrosis.

Mucoactive therapies target distinct yet interconnected aspects of the mucin barrier. Expectorants primarily enhance mucus hydration and clearance; mucolytics modify mucin polymer structure and redox balance; mucoregulators restore physiological mucin composition and suppress inflammation; and mucokinetic agents facilitate mucus transport through ciliary and cough-dependent mechanisms. While certain established agents, such as N-acetylcysteine, carbocysteine, dornase alfa, and ambroxol, demonstrate pleiotropic effects that extend beyond mucus rheology to include antioxidant, anti-inflammatory, and anti-infective actions, their clinical efficacy varies across disease phenotypes and stages, underscoring the need for personalized therapeutic strategies.

Emerging molecular targets, including NF-κB-dependent regulation of MUC5AC, calcium-activated chloride channels (CLCA-1), MARCKS-mediated mucin exocytosis, purinergic signaling pathways, and NO/cGMP signaling, offer promising avenues for the development of next-generation mucoactive agents that directly address the pathogenic drivers of mucus hypersecretion and airway remodeling. In parallel, non-pharmacological interventions such as respiratory rehabilitation and airway clearance techniques remain essential components of comprehensive management, although standardization of protocols and outcome measures is urgently required.

In summary, advancing our understanding of mucin biology at the intersection of innate immunity, inflammation, and airway mechanics is critical for the rational design of both pharmacological and non-pharmacological mucoactive therapies. Future treatment paradigms should move beyond nonspecific mucus clearance toward targeted modulation of mucin synthesis, structure, and immune function, tailored to disease mechanisms, clinical phenotypes, and patient-specific factors.

## Figures and Tables

**Table 1:** Classification of mucoactive drugs and their mechanisms of action, clinical indications, and major limitations.

Mucoactive Drugs		Mucoactive Properties	Clinical Indications	Major Limitations
Expectorants	Hypertonic saline (3–7%)	Stimulation of airway surface hydration Facilitation of sputum expectoration	Improvement of airway hydration and mucociliary clearance; mainly used in cystic fibrosis and selected cases of bronchiectasis.	May cause cough or bronchospasm; limited tolerability in airway hyperresponsiveness; evidence outside cystic fibrosis remains limited.
	Guaifenesin	Stimulation of bronchial secretion Reduction in mucus viscosity Facilitation of expectoration	Expectorant for symptomatic relief of productive cough and facilitation of mucus clearance.	Limited evidence in chronic airway diseases; mild gastrointestinal adverse effects.
	Mannitol	Osmotic airway surface hydration Facilitation of sputum expectoration	Osmotic agent enhancing airway hydration and mucociliary clearance; used in cystic fibrosis and selected patients with bronchiectasis.	May cause cough and bronchospasm; requires bronchial tolerance testing before initiation.
	Uridine triphosphate and adenosine triphosphate	Purinergic stimulation of airway secretion Activation of mucociliary clearance Facilitation of expectoration	Purinergic agonists stimulate epithelial ion and water secretion and enhance mucociliary clearance in cystic fibrosis and bronchiectasis.	Limited clinical availability and regulatory approval; short duration of action; clinical evidence remains limited.
Mucolytics	N-acetylcysteine	Disruption of mucin disulfide bonds with decreased mucus viscosity	Mucolytic and antioxidant used in chronic productive cough and mucus hypersecretion, including COPD.	Variable clinical efficacy; gastrointestinal intolerance; potential bronchospasm with inhaled formulations.
	Erdosteine	Reduction in mucus viscosity through thiol-mediated disruption of mucin disulfide bonds	Mucolytic with antioxidant and anti-inflammatory properties used in chronic bronchitis and COPD.	Limited data in diseases other than COPD; mild gastrointestinal adverse effects.
	Heparin	Reduction in mucus viscosity and airway inflammation via interaction with mucin glycoproteins and inflammatory mediators	Investigational therapy targeting mucus viscosity and airway inflammation in chronic airway diseases.	Limited clinical evidence; lack of standardized protocols; potential bleeding risk.
	Dornase alfa	Degradation of extracellular DNA in mucus, reducing viscosity and promoting clearance	Recombinant DNase that reduces mucus viscosity by degrading extracellular DNA; standard therapy in cystic fibrosis.	Limited efficacy and potential harm in non-cystic fibrosis bronchiectasis; high cost.
Mucoregulators	Carbocysteine	Regulation of mucin composition and secretion, reducing mucus hypersecretion and improving clearance	Regulates mucus composition and reduces sputum viscosity in chronic airway diseases, particularly COPD.	Evidence mainly from long-term therapy studies; gastrointestinal adverse effects.
	Anticholinergic agents	Inhibition of cholinergic-mediated mucus secretion, reducing airway hypersecretion	Bronchodilation and reduction in cholinergic-mediated mucus secretion in obstructive airway diseases.	Limited direct effects on mucus clearance; anticholinergic adverse effects.
	Glucocorticoids	Suppression of airway inflammation and regulation of mucin expression, reducing mucus hypersecretion	Anti-inflammatory therapy for airway diseases with prominent inflammation, including asthma and selected patients with COPD.	No direct mucolytic effect; limited benefit for mucus clearance; potential systemic and local adverse effects with long-term use.
	Macrolide antibiotics	Immunomodulatory regulation of mucin expression and secretion, reducing mucus hypersecretion	Long-term anti-inflammatory and immunomodulatory therapy in chronic airway diseases with recurrent infection and mucus hypersecretion, including bronchiectasis, cystic fibrosis, and selected patients with COPD.	Risk of antimicrobial resistance with prolonged use; potential adverse effects (QT prolongation, gastrointestinal symptoms); limited direct mucolytic activity.
Mucokinetics	Bronchodilators	Improved mucus transport through airway smooth muscle relaxation and enhanced airflow	Improvement of airflow and facilitation of mucus clearance in obstructive airway diseases, including asthma and COPD.	No direct mucolytic activity; effectiveness depends on underlying airway obstruction; potential cardiovascular and systemic adverse effects.
	Ambroxol	Stimulation of mucociliary transport and enhancement of mucus clearance	Mucolytic and secretolytic agent used to enhance mucus clearance in acute and chronic respiratory diseases with productive cough, including chronic bronchitis and COPD.	Limited high-quality evidence in chronic airway diseases; generally mild adverse effects (gastrointestinal symptoms, hypersensitivity reactions).

## Data Availability

This article is a narrative review and does not report original data. No new datasets were generated or analyzed. All information is derived from previously published studies cited in the reference list.
